# Leaf carbon, nitrogen, and phosphorus ecological stoichiometry of grassland ecosystems along 2,600-m altitude gradients at the Northern slope of the Tianshan Mountains

**DOI:** 10.3389/fpls.2024.1430877

**Published:** 2024-07-29

**Authors:** Yao Wang, Zhonglin Xu

**Affiliations:** ^1^ College of Ecology and Environment, Xinjiang University, Urumqi, China; ^2^ Key Laboratory of Oasis Ecology, Xinjiang University, Urumqi, China; ^3^ Xinjiang Jinghe Observation and Research Station of Temperate Desert Ecosystem, Ministry of Education, Jinghe, China; ^4^ Institute of Desert Meteorology, China Meteorological Administration, Urumqi, China; ^5^ Technology Innovation Center for Ecological Monitoring and Restoration of Desert-Oasis, Ministry of Natural Resources (MNR), Urumqi, China

**Keywords:** North slope of Tianshan Mountains, altitude gradient, grassland, ecological stoichiometry, environmental variables

## Abstract

Ecological stoichiometry of terrestrial ecosystems has been a hot issue in current research, with intense focus on the proportional relationships of nutritional elements within plants and between plants and their environment. To clarify these relationships along continuous environmental gradients is essential for a more comprehensive understanding how plants adapt to a changing environment. In arid regions, the varying plant and soil types along altitude gradients offer a unique opportunity to examine the vertical spectrum of plant and soil ecological stoichiometry. In this study, the northern slope of the Tianshan Mountains was selected as the study area to explore the carbon (C), nitrogen (N), and phosphorus (P) ecological stoichiometric characteristics of herbaceous plants along 900-m–3,500-m altitude gradients. We also investigated the variation of ecological stoichiometric characteristics among different grassland types. The results indicated that the mean C, N, and P in leaf of grassland were 342.95 g·kg^−1^–557.73 g·kg^−1^, 6.02 g·kg^−1^–20.97 g·kg^−1^, and 0.71 g·kg^−1^–3.14 g·kg^−1^, respectively. There was no significant change in leaf carbon content along the elevation gradient, and the highest and lowest leaf C concentrations were in the upland meadow and the semidesert grasslands. Both N and P concentrations obtained their highest value in the meadow steppe. The P concentration gradually increased in desert and semidesert grasslands and reached the highest value in the meadow steppe, and then decreased to the lowest value in the upland meadow and subsequently increased in the alpine meadow. The ranges of the C:N ratio, C:P ratio, and N:P ratio were 16.36–155.53, 109.36–786.52, and 2.58–17.34, respectively. Due to fluctuations in the P concentration, the C:P ratio and N:P ratio reached the lowest value in the meadow steppe and obtained their highest value in the upland meadow. Redundancy analysis showed that temperature was the dominant factor affecting the C, N, and P ecological stoichiometry of herbaceous plants, followed by soil organic carbon, mean annual precipitation, soil pH, and soil electrical conductivity. Corresponding results could enhance predictive models of nutrient cycling and ecosystem responses to climate change, particularly in arid and semiarid regions.

## 1 Introduction

Ecological stoichiometry studies the balance of multiple chemical elements and energy in biological systems during ecological interactions and processes (Hessen et al., 2013; Wang et al., 2021). Its core focuses on the interactions of elements in life processes and biogeochemical cycles, investigating the stoichiometric relationships and patterns of elements, particularly carbon (C), nitrogen (N), and phosphorus (P) in ecological processes. Carbon forms the backbone of organic matter, while nitrogen and phosphorus are fundamental components of enzymes, genetic material, and cell structures. Organisms require strict elemental ratios to catalyze metabolic reactions and synthesize essential components of life, including proteins, adenosine triphosphate ATP, and structural compounds. By simplifying complex ecological processes to the quantitative relationships and dynamic balances between elemental compositions, ecological stoichiometry unifies research findings across different levels (individual, population, community, ecosystem) from the perspective of elemental ratios (Du and Gao, 2021; Gu et al., 2022). Ecological stoichiometry plays a crucial role in environmental science. The stoichiometric balance (C:N:P ratio) has proven to be a powerful tool for quantitatively understanding and analyzing ecosystem equilibrium and processes (Frost et al., 2002; Cross et al., 2005; Zeng and Chen, 2005; Van De Waal et al., 2010; Hou et al., 2019; Feyissa et al., 2022). The C:N:P ratio can reflect plant growth rates, and the critical N:P value can serve as an indicator of nutrient supply status in the soil regarding plant growth, as well as reveal the allometric relationships and strategies of elemental distribution among plant organs. As a vital component of terrestrial ecosystems, grasslands play a significant role in energy flow and material cycling (Yang et al., 2019). Consequently, an in-depth exploration of grassland ecosystem stoichiometry can enhance our understanding of the global biogeochemical cycle and its influencing factors (He et al., 2008; Allen and Gillooly, 2009; Yu et al., 2011; Dijkstra et al., 2012; Heyburn et al., 2017; Li et al., 2023).

The stoichiometric characteristics of grasslands, encompassing both tropical and temperate varieties, display notable variation across different types and geographical locations (Yan et al., 2022). Tropical grasslands, predominantly found in central Africa, Australia, South America, and India, contrast with temperate grasslands that span regions such as the Tibetan Plateau, South Africa, South America, the former Soviet Union, and central North America. Research by He et al. (2008) into China’s grasslands revealed average leaf phosphorus (P) levels and nitrogen-to-phosphorus (N:P) ratios of 1.9 g·kg^−1^ and 15.3, respectively, suggesting that climatic factors play a minimal role in determining these stoichiometric values. In contrast, Song et al. (2014) examined the N:P:Si ratios in relation to climatic variables across seven Chinese grassland ecosystems and observed significant variability in N, P, and Si due to climatic influences, with N:P ratios particularly sensitive to mean annual temperature (MAT) and precipitation variations. Moreover, temperature exerted a greater impact compared to precipitation. Craine et al. (2008), through their studies in Kruger National Park, reported an average N:P ratio of 5.8, with N and P concentrations averaging 8.7 g·kg^−1^ and 1.8 g·kg^−1^, respectively, and noted the potential for N:P ratios to fluctuate based on soil nutrient status and precipitation. Utilizing structural equation modeling, Sun et al. (2019) demonstrated that solar radiation directly influences leaf N and P stoichiometry in the alpine meadows of the Tibetan Plateau through physiological processes while also highlighting the direct contributions of climatic variables and soil nutrients to leaf N and P stoichiometry. Additionally, Han et al. (2005) investigated the N and P stoichiometry of terrestrial plants across China, finding an average N:P ratio of 13.5 for grass species, with both N and P concentrations increasing alongside MAT, although without significant variation in the N:P ratio.

These investigations have enriched our understanding of the ecological stoichiometric traits and their determinants across global grassland ecosystems. However, the incongruent findings, particularly concerning the influence of environmental variables on the spatial variation of stoichiometric ratios, necessitate further research in specific grassland systems (Li et al., 2019). Among the diverse factors, altitude has garnered minimal attention. Given that altitude can significantly influence a variety of environmental conditions, such as temperature, precipitation, potential evapotranspiration, humidity, and the length of the growing season, as well as plant nutrient use efficiency, a thorough examination of stoichiometric ratios and the role of altitude in shaping these ratios is imperative. Current findings on this matter are fragmented and inconsistent. For instance, Reich and Oleksyn (2004) observed an increase in leaf N concentration with altitude across 1,280 plant species globally, while Tan and Wang (2016) reported an increase in leaf N concentration at altitudes below 2,350 m, with concentrations declining above this threshold. Furthermore, Reich and Oleksyn (2004) noted an altitude-induced increase in P concentration, in contrast to Ren et al. (2017), who documented fluctuating P concentrations along an altitude gradient. These disparate outcomes underscore the need for more systematic studies to understand the stoichiometric responses to altitude comprehensively.

The Tianshan Mountain, situated in the arid regions of Central Asia, exhibit a significant hydrothermal gradient, ranging from desert ecosystems at lower altitudes to alpine meadows at higher altitudes. This gradient provides a natural laboratory for examining how environmental factors such as temperature, precipitation, and soil properties influence the stoichiometric relationships of carbon (C), nitrogen (N), and phosphorus (P) in herbaceous plants. Currently, corresponding studies in the Tianshan Mountains have primarily concentrated on the ecological and physiological adaptations of plants to arid conditions and the impact of climate change on vegetation dynamics. For example, Chen et al. (2018) explored the ecological stoichiometry and interrelationship between litter and soil under seasonal snowfall in Tianshan Mountain, while Zhang et al. (2023) investigated the fine-root soil stoichiometry of *Picea schrenkiana* and its correlation with soil environmental factors under different nitrogen input levels in the Tianshan Mountains. Wang et al. (2022) studied spatial prediction models for soil stoichiometry in complex forest terrains. However, these studies mainly focused on forest and desert ecosystems, and research on the ecological stoichiometry of herbaceous plants within the Tianshan Mountain area is scarce, which constrains our comprehensive grasp of the biogeochemical cycling in this region. To address this gap, we collected leaves of herbaceous plants along an altitude range from 900 m to 3,500 m on the northern slope of the Tianshan Mountains, analyzing their C, N, and P concentrations and stoichiometric ratios. This study is dedicated to examining the variations in the C:N:P stoichiometric characteristics of grassland ecosystems in the mountainous areas of Central Asia and exploring the influence of altitude on these variations. The findings from this work contribute to a more nuanced understanding of the ecological stoichiometry and biogeochemical cycles in typical grassland ecosystems.

## 2 Materials and methods

**Figure 1:**
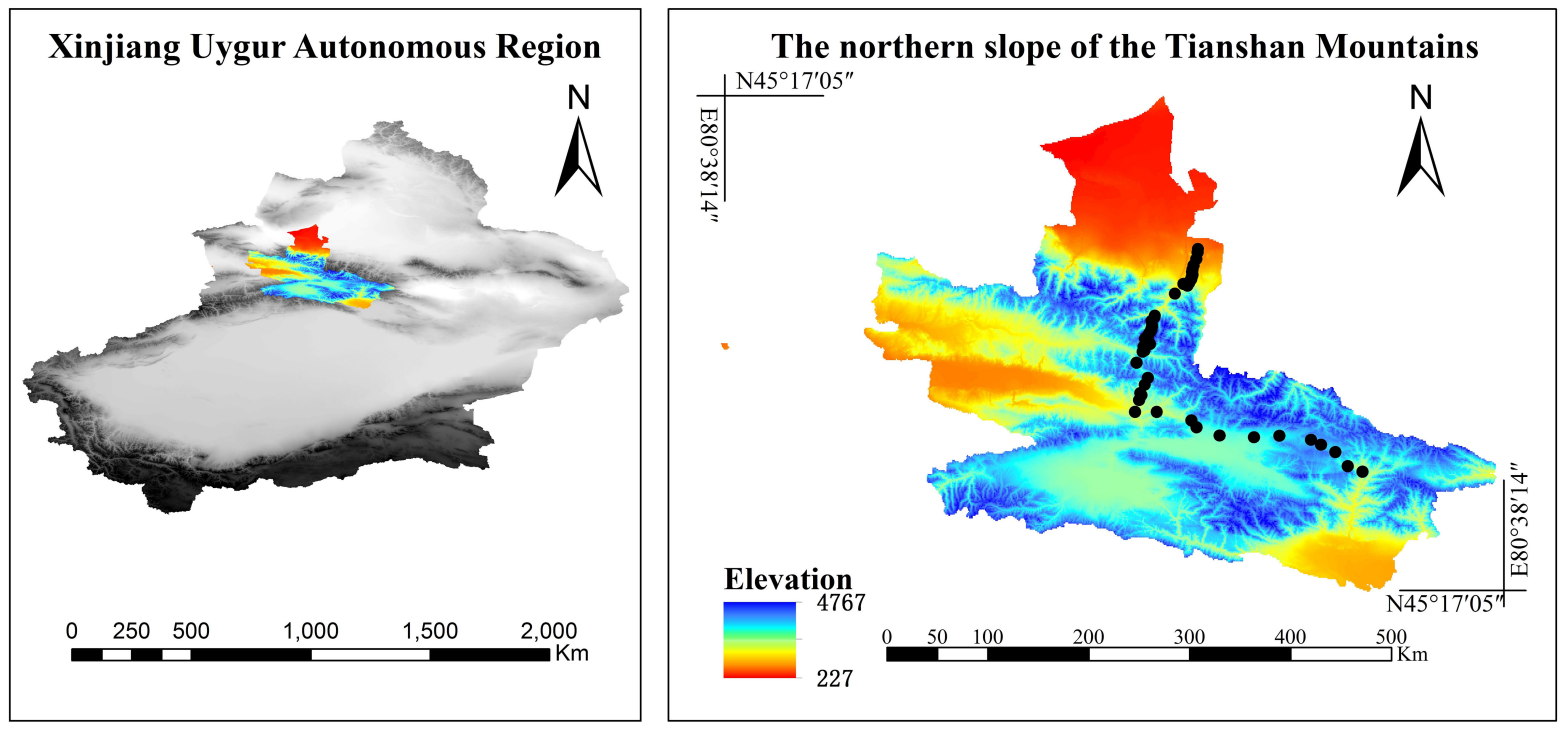
Study area.

The Tianshan Mountains, positioned at the heart of Eurasia, extend from eastern Kazakhstan to the Xinjiang province of western China, showcasing a quintessential and complete mountainous vegetation zone characteristic of the arid regions of central Asia (**Figure 1**). The sampling transects selected for this investigation is situated on the northern slope of the Tianshan Mountains, spanning an altitude range from 900 m to 3,500 m. The transect experiences a MAT ranging from −10°C to 8°C and a mean annual precipitation (MAP) between 110 mm and 630 mm, with the highest precipitation levels occurring in May and June and the lowest in February, according to Yuan and Wei (2006). Precipitation patterns vary spatially, peaking in the mid-mountain zone. According to Luo (1993), the grassland types along the altitude gradient, ascending from lower to higher altitudes, can be categorized into desert (900 m–1,200 m), semidesert grasslands (1,200 m–1,700 m), meadow steppe (1,700 m–2,000 m), upland meadow (2,000 m–2,600 m), and alpine meadow (2,600 m–3,500 m). Desert is characterized by sparse vegetation and low herb diversity. Species adapted to extreme drought conditions, such as *Haloxylon Bunge*, *Salsola* spp., *Sarcozygium Bunge*, and *Caragana Fabr*., dominate this zone. Herbe diversity of semidesert grasslands increases slightly with species like *Stipa* spp., *Agropyron* spp., and *Caragana* spp. becoming more prevalent. Meadow steppe shows a significant increase in herb diversity with species such as *Leymus chinensis*, *Festuca* spp., and various forbs. Upland meadow is rich in herbaceous species; this zone includes *Kobresia* spp., *Poa* spp., and numerous flowering plants. Herb diversity peaks in alpine meadow with a mix of grasses, sedges, and forbs like *Kobresia pygmaea* and *Carex* spp.

### 2.1 Sample collection and laboratory analysis

Leaf samples were collected from July to August 2023, spanning an altitude gradient from 900 m to 3,500 m across the Tianshan Mountains. To ensure a comprehensive sampling, 27 plots with resolution of 20 m × 20 m were established at 100-m-altitude intervals. Within each plot, the geographic coordinates and altitude were accurately determined using a global positioning system. Subsequently, five 1 m × 1 m quadrats were randomly selected for harvesting fresh leaves from the herbaceous plants. This systematic approach was designed to capture the variability in ecological stoichiometry across diverse grassland types, including desert, semidesert grasslands, meadow steppe, upland meadow, and alpine meadow. We employed a species richness-based sampling strategy to capture the diversity and ecological variability of herbaceous plant communities along the altitude gradient on the northern slope of the Tianshan Mountains. At each sampling site, we conducted a preliminary survey to assess the species richness, identifying all herbaceous species present. The number of leaf samples collected for each species was proportional to their abundance, ensuring adequate representation of both common and less common species. For each identified species, mature, healthy leaves were consistently collected from multiple individuals to ensure representativeness and minimize bias. The collected samples were then securely stored in paper envelopes and appropriately labeled. Following collection, a total of 81 leaf samples were prepared for analysis. These samples were dried in an oven at 65°C until they reached a constant weight and subsequently ground into a fine powder using a mortar. The concentrations of total C, N, and P in the leaves were quantified using the acid-dichromate FeSO_4_ titration method (Schumacher, 2002), the Kjeldahl digestion method (McKenzie and Wallace, 1954), and the H_2_SO_4_-HClO_4_ fusion method (Helmke and Sparks, 1996), respectively. The stoichiometric ratios of C:N, C:P, and N:P were then calculated on a mass basis, providing essential data for understanding the nutritional dynamics within the grassland ecosystem of the Tianshan Mountains.

### 2.2 Data processing

The MAT and MAP values were sourced from the WorldClim 2 datasets (Fick and Hijmans, 2017), providing a comprehensive and high-resolution climate dataset for our study area. Soil physical and chemical parameters were retrieved from the Resources and Environment Scientific Data Center of the Chinese Academy of Sciences, available online at http://www.resdc.cn (accessed on 08 June 2023). This data center offers a valuable repository of environmental and ecological data pertinent to China. To explore the relationships between various environmental factors and stoichiometric data, Pearson correlation analysis was employed. This statistical method facilitated the identification of significant correlations, enabling a deeper understanding of the interplay between climate, soil characteristics, and plant nutritional dynamics along the altitude gradient in the Tianshan Mountains.

Redundancy analysis (RDA) is an advanced multivariate technique that synergizes regression analysis with principal component analysis to assess the impact of explanatory variables on response variables. In our analysis, leaf C, N, and P stoichiometry served as the response variables, while soil electrical conductivity (EC), soil pH, soil organic carbon (SOC) concentration, MAT, and MAP were the explanatory variables. For different grassland types (desert, semidesert grassland, meadow steppe, upland meadow, and alpine meadow), we first calculated the mean values of stoichiometric characteristics. Subsequently, we applied RDA to elucidate the relationships between these stoichiometric traits and the environmental variables. A crucial step in RDA is evaluating the variance inflation factors (VIFs), which indicate the extent to which correlations among explanatory variables inflate the variance of the canonical coefficients, potentially destabilizing the regression model. Following Ter Braak’s (Ter Braak, 1990) recommendation, we considered variables with VIFs exceeding 20 as unsuitable for analysis. Additionally, we conducted a detrended correspondence analysis (DCA) to determine the length of the gradient (LGA). For these analyses, we utilized Canoco software (version 5.0), as recommended by Ter Braak and Smilauer (2002), to ensure a robust and insightful examination of the ecological stoichiometry within the leaf samples from the Tianshan Mountains.

## 3 Results

### 3.1 Correlation between C, N, and P ecological stoichiometric characteristics of leaves

**Figure 2:**
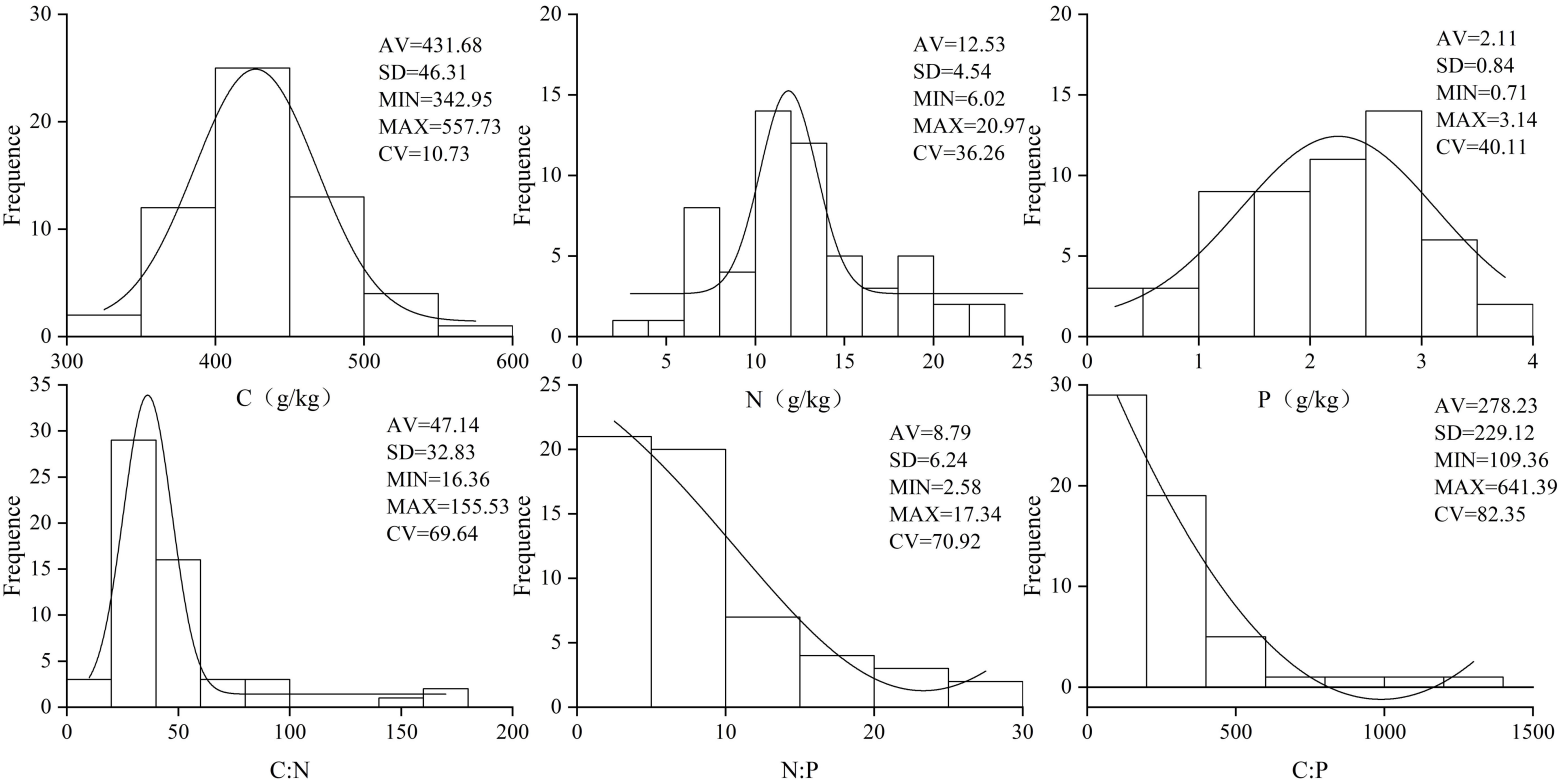
C, N, and P ecological stoichiometric characteristics of leaves (AV, SD, MIN, MAX, CV denote average, standard deviation, minimum, maximum, and coefficient of variation, respectively).

Leaf C, N, and P concentrations and C:N, N:P, and C:P ratios exhibited large variations, primarily ranging 342.95 g·kg^−1^–557.73 g·kg^−1^ for C, 6.02 g·kg^−1^–20.97 g·kg^−1^ for N, 0.71 g·kg^−1^–3.14g·kg^−1^ for P, 16.36 g·kg^−1^–155.53 g·kg^−1^ for C:N, 2.58 g·kg^−1^–17.34 g·kg^−1^ for N:P, and 109.36 g·kg^−1^–641.39 g·kg^−1^ for C:P. The corresponding average values were 431.68 g·kg^−1^, 12.53 g·kg^−1^, and 2.11 g·kg^−1^ and 47.14, 8.79, and 278.23, respectively. Accordingly, the coefficients of variation were 10.73%, 36.26%, 40.11%, 69.64%, 70.92%, and 82.35% (**Figure 2**).

**Figure 3:**
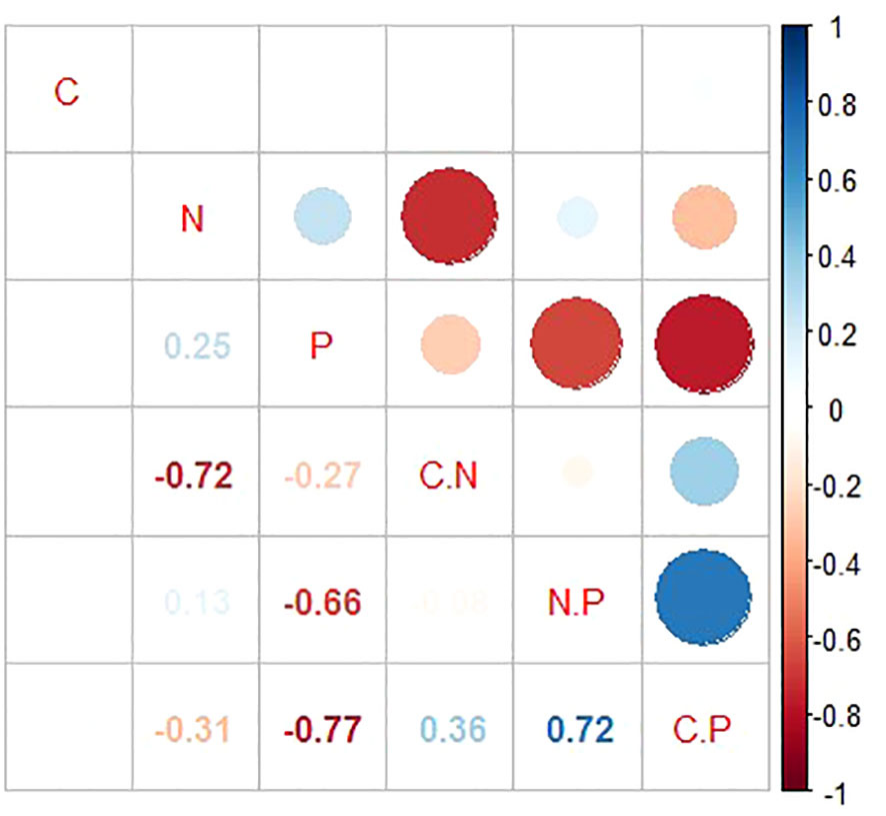
C, N, and P ecological stoichiometric characteristics correlation thermogram (The size and color intensity of the circles represent the strength and direction of the correlations, with blue indicating positive correlations and red indicating negative correlations.).

There was not significant correlation among C, N, and P concentrations in the leaf samples as indicated in **Figure 3**. Notably, the N concentration exhibited a negative correlation with both C:N and C:P ratios, evidenced by correlation coefficients of −0.72 (p < 0.01) and −0.31 (p < 0.05), respectively. Similarly, the P concentration was found to have significant negative correlations with C:N, C:P, and N:P ratios, with correlation coefficients of −0.27, −0.77, and −0.66, respectively, highlighting a strong inverse relationship between the P concentration and these stoichiometric ratios. Moreover, significant positive correlations were observed between C:N and C:P ratios, as well as between C:P and N:P ratios, suggesting a coordinated variation among these stoichiometric ratios in the leaf samples.

### 3.2 Variation of C:N:P ratios among different grassland types

**Figure 4:**
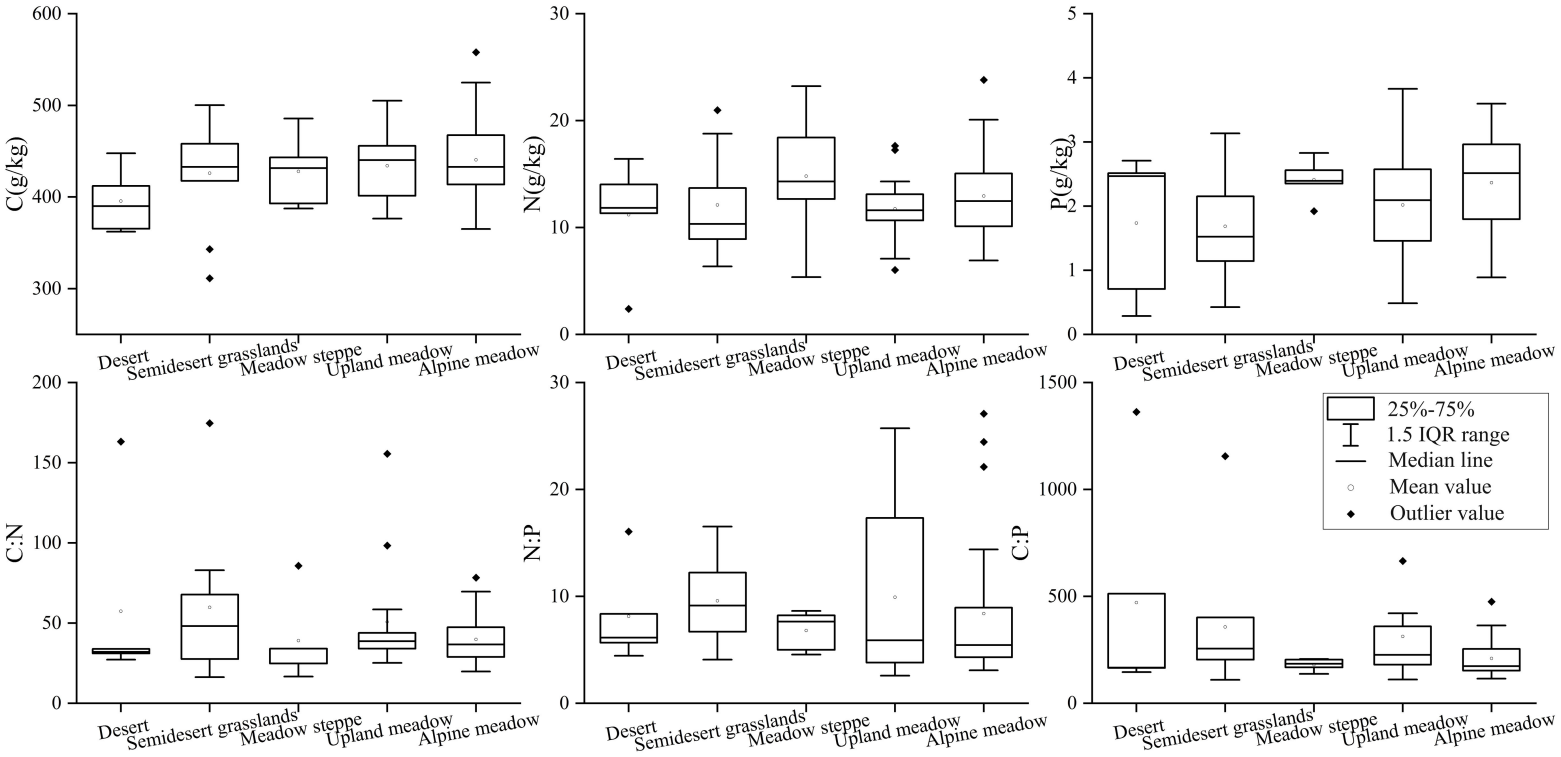
C, N, and P ecological stoichiometric characteristics among different grassland types (each dot represents a sample plot; these plots indicate the complex and variable nature of nutrient concentrations and stoichiometric ratios in grassland ecosystems along the altitude gradient).

Among different grassland types, the C concentration remained relatively stable, exhibiting the highest values in alpine meadows and the lowest in semidesert grasslands. The N concentration displayed a trend of initial increase followed by a decrease, peaking in meadow steppes. The P concentration showed a gradual increase starting from desert environments, reaching its zenith in meadow steppes, where it then stabilized with minimal fluctuations. The ratios of C:N and C:P were relatively stable across the different grassland types, with semidesert grasslands and meadow steppes showing the highest and lowest values, respectively. Interestingly, the C:P ratio was lowest in deserts and peaked in upland meadows. Conversely, the N:P ratio exhibited variability across the grassland types, with its highest and lowest values observed in upland meadows and meadow steppes, respectively, as depicted in **Figure 4**.

### 3.3 Response of leaf C, N, and P stoichiometry to environmental factors

The results from the redundancy analysis (RDA) demonstrated that the cumulative explanatory power of the first two axes accounted for 99% of the total variation. This significant level of explained variation indicates that these axes are highly effective in depicting the relationships between the stoichiometric characteristics of the samples and the environmental factors under investigation. Thus, the first two axes of the RDA provide a comprehensive view of how stoichiometric patterns are influenced by the surrounding environmental conditions, underscoring the utility of RDA in ecological research for understanding complex interactions between biotic traits and abiotic factors (**Table 1**).

**Table 1:** RDA results of stoichiometric characteristics of herb C, N, and P.

Ordering axis	Axis I	Axis II		Axis III	Axis IV
Stoichiometric interpretation (%)	9	1.3	0		0
Correlation between stoichiometric characteristics and soil environmental factors (%)	0.338	0.265	0.188		0.252
Cumulative interpretation of stoichiometric characteristics (%)	9	10.3	10.3		10.3
Stoichiometry-cumulative interpretation of soil environmental factor relationships (%)	86.4	99	99.4		100
Canonical eigenvalue	0.104				
Total eigenvalue	1				

**Figure 5:**
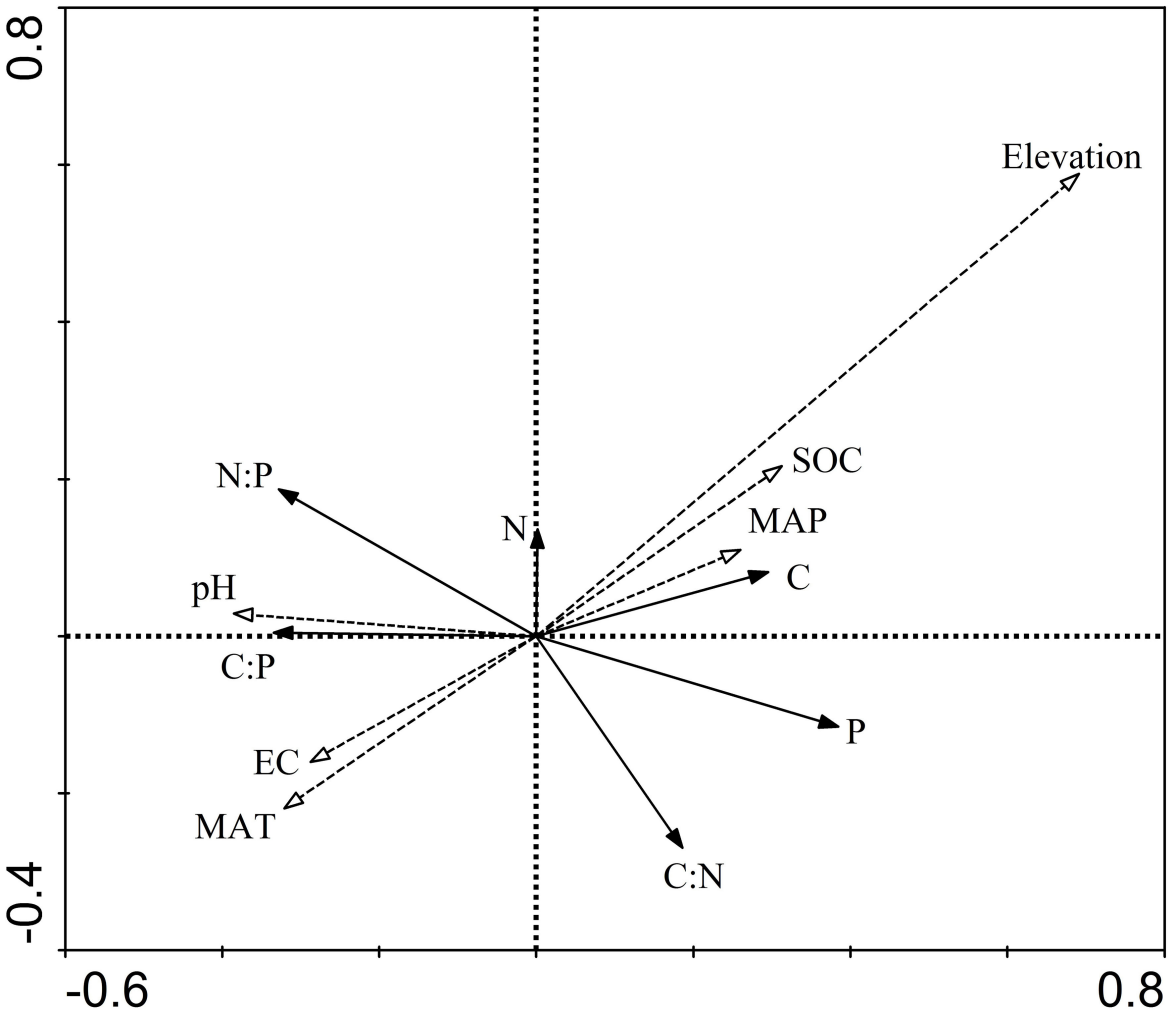
Bidimensional ordering chart of the RDA of relationships of stoichiometric characteristics of leaf C, N, and P; this figure highlights the dominant role of elevation and temperature in shaping the nutrient dynamics within the studied grassland ecosystems.

Among different environmental variables, MAT emerged as the most influential in explaining variations in stoichiometric characteristics, as illustrated in **Figure 5**. C, N, and P concentrations were found to be positively correlated with SOC and MAP and negatively correlated with EC and MAT. Soil pH exhibited a positive correlation with N concentration, C:P ratio, and N:P ratio while showing a negative correlation with C and P concentrations and C:N ratio. It is important to note that the RDA biplot (**Figure 5**) reveals correlations between environmental factors and stoichiometric characteristics but does not quantify the contribution of these factors to the characteristics. Through the application of a forward screening method and the Monte Carlo test, the environmental factors were ranked in terms of their importance as follows: MAT > SOC > MAP > soil pH > EC. The significant influence (p < 0.05) of MAT underscores its importance in affecting stoichiometric characteristics. In contrast, soil pH and EC were found to have limited explanatory power for the variations in the stoichiometric characteristics of plant leaves (**Table 2**), highlighting the critical roles of climate factors in shaping nutrient dynamics within grassland ecosystems.

**Table 2:** Explanation quantity and significance of environmental factors.

Environmental factors	Importance sequencing	F	P
MAT	1	10.30	0.042
SOC	2	10.28	0.06
MAP	3	11.28	0.065
pH	4	10.29	0.706
EC	5	3.56	0.528

## 4 Discussions

Our findings reveal that the leaf carbon (C) concentration in our study, averaging 431.68 ± 46.31 g·kg^−1^, was lower compared to the global average for terrestrial plants reported by Elser et al. (2000) yet aligned with observations of grassland species within China documented by He et al. (2006). This lower leaf C concentration in the arid environments of our study area can be attributed to reduced stomatal conductance under water scarcity, which hampers the carbon sequestration process, leading to lower C concentrations than those observed in more humid regions (Yu and Wang, 1998). The nitrogen (N) concentration in our samples, at 12.53 ± 4.54 g·kg^−1^, contrasts with the higher concentrations reported in herbaceous plants both globally (Reich and Oleksyn, 2004) and within China (Han et al., 2005) but is consistent with findings from the Alexa desert (Zhang et al., 2016), likely due to the low N concentration in the soil (Wang and Yu, 2008; Xie et al., 2016). The phosphorus (P) concentration, at 2.11 ± 0.84 g·kg^−1^, exceeded that found in halophytes of northwestern China (Wang et al., 2015), typical deserts in northern China (Shi et al., 2017), and matched results from northeastern China (Huang et al., 2012). The higher P concentration in herbaceous plants compared to shrubs and trees (Liu et al., 2017), along with an observed gradient of increasing soil P concentration from humid to arid and semiarid regions in China (Lu and Tian, 2017), might explain the relatively high leaf P concentrations in our study area.

The C:N ratio of 16.36–155.53, with an average of 47.14 ± 32.83, was found to be higher than both the global average (Elser et al., 2000) and averages for herbaceous plants in northwest China (Wang et al., 2015). The C:P ratio of 278.23 ± 229.12 in our study was below the global average, possibly indicating higher P use efficiency as suggested by Jia (2015). The average N:P ratio of 8.79 ± 6.24 was lower than that reported for global terrestrial plants and herb species in other parts of China (Han et al., 2005; Abdurahman et al., 2015; Zhang et al., 2016; Shi et al., 2017), suggesting that herbaceous plants on the northern slope of the Tianshan Mountains are likely constrained by N availability, as indicated by an N:P ratio below the threshold of 14, which denotes N limitation (Elser et al., 2001). This aligns with findings that plants in temperate regions often face significant N constraints (Sha et al., 2011).

The observed variation in nitrogen (N) concentrations along the altitude gradient in our study, characterized by an increase–decrease trend, parallels findings from the Gongga Mountain as reported by Tan and Wang (2016). This pattern may be attributable to the interplay of two mechanisms: the Temperature-Physiological Hypothesis (TPPH) (Feng et al., 2003) and the Biogeochemical Hypothesis (BH) (Ohtaka and Nishino, 1999). The TPPH posits that lower temperatures at higher altitudes impede N-involved enzyme activities, thus dampening biochemical reactions (Feng et al., 2003). In response, herbaceous plants may augment their N concentration to counterbalance the reduced biochemical activity, leading to an elevation-correlated increase in leaf N concentration (Su et al., 2022). Conversely, the BH suggests that colder conditions at high altitudes limit soil nutrient mineralization, curtailing plant nutrient uptake and leading to a reduction in plant N concentration with altitude (Ohtaka and Nishino, 1999). In our study, the dominant mechanism shifts along the altitude gradient: below 2,000 m, TPPH predominantly influences N concentration, while above this threshold, BH becomes more significant. Similarly, the phosphorus (P) concentration in leaves exhibited an upward trend with increasing altitude up to 2,000 m. This rise could be driven by enhanced precipitation and soil moisture at higher altitudes, which in turn accelerates soil parent material weathering rates (Zhou, 2015). However, the decline in P concentration observed from 2,000 m to 2,600 m and the subsequent fluctuation above 2,600 m may result from a combination of leaching effects and physiological adaptations. To mitigate metabolic inhibition induced by lower temperatures, plants may need to sustain high concentrations of N and P (Feng et al., 2003; Reich and Oleksyn, 2004), reflecting a complex balance between environmental factors and plant physiological responses across the altitude gradient. Our findings on the trends in N and P concentrations along the altitude gradient have several important implications for grassland management and conservation in arid mountainous regions. For instance, areas identified as having lower N or P concentrations may benefit from site-specific nutrient supplementation to enhance plant growth and ecosystem productivity. In addition, conservation efforts should aim to preserve these natural nutrient balances to support diverse plant communities and their associated fauna. In degraded areas where nutrient imbalances are observed, restoration efforts can focus on reestablishing native plant species that are adapted to the local nutrient conditions, thereby enhancing ecosystem resilience.

Throughout the protracted evolution of photosynthesis and mineral metabolism, plants have developed the capacity to adapt to environmental fluctuations. The variations in the concentrations of C, N, and P within plants, along with changes in their ecological stoichiometric ratios, are indicative of the plants’ responses to environmental shifts as well as their physiological and biochemical adaptability (Tian et al., 2007; Shi et al., 2013; Li et al., 2023; Bai et al., 2024). The findings from this study reveal that environmental factors exert diverse impacts on the C, N, and P stoichiometric characteristics of herbaceous plants, aligning with insights from prior research (Reich and Oleksyn, 2004; Tan and Wang, 2016). Among these factors, MAT emerges as the most significant, followed SOC, MAP, soil pH, and EC. It has been posited in previous studies that temperature plays a crucial role in determining the concentrations of C, N, and P and their stoichiometric ratios (Han et al., 2005; Lu et al., 2016; Xie et al., 2016; Bai et al., 2024). Typically, as altitude increases and temperature decreases, plants may augment their elemental concentrations to compensate for reduced photosynthetic rates (Feng et al., 2003; Yang et al., 2023). Furthermore, changes in precipitation and atmospheric pressure along the altitude gradient can influence the physiological traits of leaves, thereby affecting elemental concentrations (Warren and Adams, 2004; Chen et al., 2024). Additionally, related research has found that the leaf C:N ratio remains stable across three different climatic regions: the temperate grasslands of Inner Mongolia, the alpine grasslands of Tibet, and the mountain grasslands of Xinjiang. However, significant differences exist among coexisting species and different vegetation types. It is believed that life forms and genus characteristics account for more than 70% of the variation in the C:N ratio while seasonal average temperature and precipitation explain only 3%. Therefore, at the biosphere scale, temperature, and precipitation may affect the leaf C:N ratio by altering plant species composition (Yang and Wang, 2011). In the same study regions, climatic variables have almost no direct correlation with the leaf N:P ratio. Seasonal precipitation and temperature explain only 2% of the variation, whereas inter-site differences and intra-site systematic variations explain 55% and 26% of the N:P ratio variation, respectively (He et al., 2008). Therefore, different limiting factors in growth regulation may lead to variations in the C:N, C:P, and N:P ratios observed in related studies and their responses to environmental conditions. The indirect effects of altitude also constrain soil microbial activity and the rate of soil organic matter decomposition, which in turn limits root nutrient absorption and utilization, impacting plant tissue elemental concentrations (Chen et al., 1996; Liao et al., 2024). In addition, the Tianshan Mountains exhibit significant microhabitat variability due to differences in soil types, moisture availability, and microclimatic conditions within short spatial scales. This heterogeneity can mask broader altitude trends as localized factors exert strong influences on nutrient concentrations. Simultaneously, herbaceous plants in this region may possess high physiological plasticity, allowing them to maintain stable nutrient concentrations despite varying environmental conditions. Such adaptations can result in consistent stoichiometric ratios across different altitudes. Previous research has underscored the critical impact of precipitation on the stoichiometry of C, N, and P concentrations in herbaceous plants (Huang et al., 2012; Hong et al., 2013). However, our study did not find a significant impact of MAP, possibly because plants can modulate nutrient usage to adapt to moisture constraints (Bai, 2013; Qin et al., 2024). Since the redundancy analysis (RDA) highlighted predominance of MAT over MAP, it suggests that temperature constraints on nutrient absorption and utilization are more significant than moisture constraints, reflecting the adaptive strategies of plants to their environment and their inherent robust internal stability (Yao and Zhu, 2006; Yu et al., 2024).

## 5 Conclusions

This research delves into the ecological stoichiometry of herbaceous plants across the altitude gradient of the Tianshan Mountains’ northern slopes, revealing a complex narrative of plant adaptation to environmental variables. The study identifies that carbon (C) concentrations in plant tissues remain stable across varying altitudes, while nitrogen (N) and phosphorus (P) display more dynamic patterns: increasing and then decreasing for N and gradually ascending before peaking and declining for P. These elemental fluctuations are not merely random; they are intricately linked to the environmental gradients of temperature, with soil properties also playing a role, albeit secondary to the climatic influences. The corresponding findings underscore the critical role of climatic variables in influencing the stoichiometric balance of C, N, and P within plant communities. Temperature, in particular, emerges as a key determinant, influencing nutrient uptake and utilization, thereby affecting plant growth and ecological balance. This suggests a nuanced interaction between plants and their environment, where physiological adaptations enable survival and growth despite the challenges posed by altitude and climatic variability. By highlighting the dominant impact of altitude and temperature on plant nutrient dynamics, this study contributes valuable insights into the adaptive strategies of grassland ecosystems in mountainous regions. It underscores the necessity for integrated ecosystem management approaches that consider the influence of climatic gradients on plant nutrient dynamics. In the context of global climate change, understanding these ecological stoichiometry patterns is crucial for preserving the biodiversity and functionality of these vital ecosystems, ensuring their resilience and sustainability for the future. To further enhance our understanding of ecological stoichiometry in mountainous grassland ecosystems, we recommend the following areas for future research: firstly, conducting year-round sampling to capture seasonal variations in nutrient concentrations and stoichiometric ratios. This would provide a more comprehensive understanding of how stoichiometric characteristics change over different seasons and under varying climatic conditions. Second is investigating the fine-scale variability in soil and microclimatic conditions within each elevational band. This would help to better understand the influence of local environmental factors on nutrient dynamics and plant physiology. Third is exploring the role of biotic interactions, such as competition and herbivory, in shaping nutrient dynamics. Understanding these interactions could reveal important ecological processes that influence stoichiometric patterns.

## Data availability statement

The original contributions presented in the study are included in the article/supplementary material. Further inquiries can be directed to the corresponding authors.

## Author contributions

YW: Conceptualization, Funding acquisition, Methodology, Visualization, Writing – review & editing. ZX: Conceptualization, Funding acquisition, Investigation, Methodology, Project administration, Visualization, Writing – original draft.
